# A comparative analysis of readability, quality, and reliability in large language model outputs pertaining to knee osteoarthritis queries

**DOI:** 10.1371/journal.pone.0353355

**Published:** 2026-07-29

**Authors:** Erdem Maraşlı, Erkan Ozduran, Volkan Hancı

**Affiliations:** 1 Aydın State Hospital, Physical Medicine and Rehabilitation, Pain Medicine, Aydin, Turkey; 2 Sivas Numune Hospital, Physical Medicine and Rehabilitation, Pain Medicine, Sivas, Turkey; 3 Dokuz Eylul University, Anesthesiology and Reanimation, Critical Care Medicine, Izmir, Turkey; Ondokuz Mayıs Üniversitesi: Ondokuz Mayis Universitesi, TÜRKIYE

## Abstract

This study aims to comparatively examine the readability, accuracy, and quality of responses provided by artificial intelligence (AI)-based chatbots such as Perplexity, ChatGPT-5, and Gemini to questions about knee osteoarthritis (KOA), which accounts for approximately four-fifths of the global osteoarthritis (OA) burden. In this study, 8 keywords were determined by excluding repetitive, irrelevant or synonymous ones from the 25 most frequently used English keywords associated with KOA based on Google Trends data, and these terms were asked as questions to three different artificial intelligence-based chatbots. The study measured readability using formulas like Coleman-Liau Index (CLI), Automated Readability Index (ARI), and Linsear Write (LW). Reliability of the information was assessed using the Journal of the American Medical Association (JAMA) benchmarks along with the modified DISCERN instrument. To determine overall content quality, the Global Quality Score (GQS) and the Ensuring Quality Information for Patients (EQIP) scale were applied. Together, these tools provided a comprehensive assessment of how understandable, reliable, and high-quality each chatbot’s responses were. The most frequently searched keywords related to OA were “osteoarthritis of knee,” “knee pain,” and “osteoarthritis knee pain.” A readability analysis of responses from three different AI-based chat systems revealed that all platforms had text levels above the Grade 6 threshold, and this difference was statistically significant (p < 0.05). Comparisons demonstrated that ChatGPT-5 produced the most readable content (FRES:45, GFOG:11.9, FKGL:9.24, CLI:14.03, SMOG:8.37, ARI:11.37, LW:7.2). However, Perplexity achieved significantly higher scores than ChatGPT-5 across all quality and reliability assessments, yielding superior median scores (DISCERN: 4, JAMA: 2, GQS: 4, EQIP: 92.8). Perplexity also outperformed Gemini in the mDISCERN reliability assessment (p = 0.001), while no significant difference in quality or reliability was found between Gemini and ChatGPT-5. No statistically significant difference was found between Gemini and ChatGPT in reliability and quality surveys. This analysis of KOA highlights significant challenges regarding the potential of popular AI chatbots for patient information. When examining readability levels, responses from these tools consistently exceed the recommended comprehensibility threshold, making it difficult for patients to absorb critical information. Furthermore, the relatively low scores recorded in reliability and content quality assessments raise significant concerns about the scientific validity and integrity of the medical information presented. Given these findings, the sufficient quality, robustness, and appropriate levels of understandability of future AI-based tools can only be ensured by the establishment and operation of an effective oversight mechanism.

## Introduction

Osteoarthritis (OA), a major cause of knee pain and physical disability, is a degenerative joint disease characterized by the degeneration and loss of articular cartilage. It can affect any joint in the body, leading to pain, stiffness, and limited mobility; this often results in a poor quality of life [1]. Knee OA (KOA) accounts for approximately four-fifths of the global OA burden, with prevalence rising with obesity and advancing age [2]. Symptomatic OA is reported in approximately 13% of women and 10% of men aged 60 and over [3]. Furthermore, the overall prevalence of knee or surrounding pain is reported to be 47% (men 44%, women 49%), with women reporting higher levels of knee pain [1]. The Global Burden of Disease study, published in 2010, identified hip and knee OA as among the largest contributors to disability [4]. In the United States (US), the overall economic burden of KOA is estimated at $27 billion annually. More than half of patients are expected to undergo total knee replacement, resulting in an estimated lifetime cost of $140,300 for a patient diagnosed with KOA [5]. The rising global prevalence of osteoarthritis between 1990 and 2019, driven primarily by aging and demographic growth, exacerbates health disparities and necessitates urgent, equitable public health strategies [6]. Current management strategies for OA include a variety of modalities, including exercise and physical therapy, assistive devices such as canes, living space modifications, patient self-management education, pharmacological pain medications, and surgical interventions, including joint replacement [7].

The penetration of Artificial Intelligence (AI) into nearly every aspect of life has made its integration into healthcare, health-related information acquisition processes, and especially diagnostic mechanisms inevitable. AI stands out not only for its potential to deliver better results, higher speed, increased accuracy, and efficiency, but also for its low cost and reduced manual effort, infrastructure, and equipment requirements [8]. These AI models, in particular, represent a shift from traditional search engines that provide information to next-generation interfaces that can provide detailed answers through reasoning and “logical inference” capabilities [9]. These user-friendly features propelled ChatGPT, one of the most popular artificial intelligence applications, to 100 million users in just 2 months after its launch in November 2022 [10]. However, because machine learning methods are not well-suited to distinguish between accurate and inaccurate data, the ChatGPT system frequently makes factual errors and provides imprecise information, often referred to as “hallucinations” in the literature [11]. The integration of AI into health information-seeking processes introduces significant risks for the general public. Because these models often operate as ‘black boxes’ and lack transparency, the inaccurate content they produce can lead to misinformed self-management decisions by patients. Furthermore, when AI-generated advice contradicts evidence-based clinical guidelines, it risks eroding the trust between patients and their healthcare providers [12]. This study addresses these challenges by systematically comparing popular AI models to identify which platforms offer the most reliable and accessible information. Another problem observed in Large Language Models is hallucinations, which tend to produce outputs inconsistent with visual content. Because these hallucinations raise serious safety and ethical concerns in practical applications due to inaccurate or misleading visual-language outputs, it is emphasized that mitigation strategies should aim not only at improving accuracy but also at reducing these potential security risks [13]. Therefore, a global regulatory alignment for AI in healthcare, similar to the voluntary AI code of conduct developed by the US-EU (European Union) Trade and Technology Council, would be beneficial to all countries, whether developing or developed [14]. Furthermore, a growing body of evidence suggests a strong correlation between online interaction and research visibility via Altmetrics, indicating that digital dissemination strategies can significantly amplify the broader impact of health-related information [15].

It has been reported that approximately half of the adult population uses the internet to obtain health-related information, and access to online health-related information among individuals aged 16–74 in Europe increased by 21% from 2010 to 2020 [16,17]. However, the concept of health literacy emerges in understanding and managing this written health information. Health literacy is the ability to understand and use health information, enabling individuals to make informed decisions. Deficits in health literacy has serious consequences, leading to poor interpretation of health data and to individuals with low literacy levels experiencing more adverse health outcomes [18]. Low health literacy leads to poorer health outcomes for individuals and also contributes to increased healthcare costs due to factors such as emergency room readmissions and inappropriate service utilization [19]. According to data from the National Library of Medicine, nearly 90% of adults—even those who are generally proficient readers—face challenges in understanding health-related information. Similarly, findings from the US Department of Education’s National Center for Education Statistics indicate that more than half (approximately 54%) of Americans between the ages of 16 and 74 read at a level below the sixth grade. Reflecting these concerns, major health organizations such as the American Medical Association (AMA), the AMA Foundation, and the National Institutes of Health (NIH) recommend that all patient education materials be written in plain language, ideally at or beneath a sixth-grade reading level, to promote accessibility and comprehension [16,20]. Therefore, Patient Education Materials (PEMs) have become an absolute necessity for health information producers because their simplified and understandable format significantly increases information comprehension in individuals with low literacy skills [18].

KOA is one of the most frequently searched health topics online, yet the accuracy, readability, and reliability of information generated by AI-based chatbots remain unclear. Despite the increasing use of ChatGPT, Gemini, and Perplexity for medical guidance, no prior study has directly compared the KOA-related outputs of these three widely used AI systems, representing a key gap in the current literature. This study aims to address that gap by systematically evaluating the readability, quality, and reliability of chatbot-generated health information on KOA. Our central hypothesis is that the readability, reliability, and content quality of KOA information will vary significantly between AI models, and that most outputs will exceed the recommended sixth-grade reading level for patient education. This study was designed as a descriptive and exploratory analysis aiming to provide a baseline assessment of AI-generated health information related to KOA. The objective was not to evaluate clinical effectiveness or patient outcomes, but rather to examine readability, quality, and reliability characteristics of chatbot-generated content under real-world usage conditions.

## Materials and methods

### Ethical authorization

This study did not involve human participants, animal subjects, or any sensitive/identifiable personal data. The data collection process was limited to the evaluation of publicly accessible, computer-generated outputs from artificial intelligence platforms (ChatGPT, Gemini, and Perplexity). Since the research analyzed exclusively non-human, publicly available digital content and involved no interaction with human subjects, it was deemed exempt from institutional ethics committee (IRB) approval [9,18]. This exemption is consistent with the established ethical frameworks for secondary data analysis and infodemiology research.

### Study design

Before starting the study, personal internet browser cookies and browsing history were completely deleted to minimize potential subjective bias. Data collection was conducted using Google’s Incognito mode, preventing the influence of previous user data on the results. The data collection process was conducted on September 1, 2025, in Turkey. All queries were executed through a local network interface without the use of a Virtual Private Network (VPN) to reflect the standard user experience within this geographic region. To ensure consistency and prevent bias, all models were queried on the same day within a 4-hour window. While the English language was used to ensure global relevance, it is noted that Large Language Model (LLM) responses may vary slightly based on regional server nodes and localized algorithms.

Search frequency and geographic distribution data for the keyword “Knee Osteoarthritis” were analyzed using the Google Trends (https://trends.google.com/) platform. In this context, global data from 2004 to the present was examined using the “most relevant” results filter, and the 25 most frequently searched keywords worldwide and the geographic interest density of users were identified and recorded [21]. The main reason for choosing this method is that the Google search engine has a high global market share among other search engines [22]. This dominant position of Google justified its use as the primary search tool to ensure access to the most reliable and comprehensive database for the study. The primary target audience of this investigation is the public seeking health information online. By utilizing keywords derived from Google Trends, the study aimed to replicate the actual online seeking behavior of patients rather than the specialized informational needs of medical professionals.

Duplicates/synonyms and non-KOA keywords were determined as exclusion criteria for our study, while KOA-related English language keywords were determined as inclusion criteria for our study. To this end, the selected keywords were posed to these AI models as queries in English [23,24]. As per the research protocol, each keyword was submitted to the chatbots separately through different user sessions, thus minimizing the risk of systematic bias that could arise from sequential processing of key terms. The responses generated by the AI models were systematically recorded and evaluated based on reliability, readability, and content quality criteria.

To provide a more detailed analysis and better define the nature of the information presented to users, the eight selected keywords were subdivided into three pre-specified functional categories: Disease Description (“osteoarthritis of knee”,”what is osteoarthritis knee); Symptomatology (or diagnostic-related information), (“knee pain”, “osteoarthritis knee pain”,”osteoarthritis knee symptoms”); and Treatment/Management (“osteoarthritis treatment knee“, “knee osteoarthritis exercises”, “knee replacement”).

All keywords and related responses generated by AI chatbots are made available via the web archive link below: https://zenodo.org/records/21163203

The dataset for this study comprises 24 distinct responses generated by three AI models (ChatGPT-5, Gemini 2.5 Flash, and Perplexity) based on eight high-frequency keywords identified through Google Trends. We confirm that all data collection and analysis methods were conducted in strict compliance with the **Terms and Conditions** and usage policies of the respective AI platforms.

The selection of ChatGPT-5, Gemini, and Perplexity was based on their global market dominance and distinct architectural approaches.To maximize reproducibility, we used the **default, free public versions** of each LLM without any customized system prompts or hyperparameter adjustments (e.g., temperature or top-p), as these are not configurable via the standard web interfaces used by the general public. This study used GPT-5, the latest and free version of ChatGPT, one of the most accessible AI platforms in terms of social accessibility, released on August 7, 2025. Verification was made by paying attention to the free version and the ChatGPT-5 version in all data collection processes. This model was chosen because it allows users from different socioeconomic backgrounds to systematically assess the content integrity and accuracy of information accessed through this technology. To broaden the scope of the comparative analysis and enhance the robustness of the findings, the free versions of the Gemini (2.5 Flash) and Perplexity (Standart Sonar model) platforms were also included in the study [23,25]. During our study, we used the free version and default mode of all three chatbots. Detailed information about the AI chatbots used is provided in the Table 1.

**Table 1:** Configuration and Feature Availability of the Large Language Models evaluated in this study.

Model	Access Type	Reasoning Mode	Web Search	Deep Research	Additional Notes
**ChatGPT 5**	Web-based (ChatGPT Free)	**Not available** (GPT-5 has no separate reasoning mode)	**Available**	**Not available for GPT-5** (Only for o-models)	All tests conducted using default settings. No manual enabling of browsing or tool integrations.
**Gemini 2.5 Flash**	Web interface (gemini.google.com)	Has reasoning capability built into its core architecture but does not offer a distinct reasoning mode toggle.	**Integrated search always ON** (Google Search is embedded)	Can perform but does not have a specific “Deep Research Mode” toggle	All tests conducted with default **Gemini 2.5 Flash model**
**Perplexity (Sonar)**	Web-based (perplexity.ai)	Although reasoning mode is toggleable, utilized in default mode. In this mode, reasoning mode is off.	**Always ON**	**Perplexity’s Sonar default mode does not include Deep Research.**	All tests conducted with default Sonar mode without enabling “Deep Search” or Reasoning Mode.

### Readability analysis of texts

In this study, the online platform available at http://readabilityformulas.com/ was used to systematically assess the readability level of keyword responses generated by AI chatbots. Images were removed from AI chatbot responses and long/short output texts, regardless of length, were copied and pasted to this web address to measure readability To ensure a robust and multidimensional assessment of text complexity, seven distinct readability metrics were employed (Flesch–Kincaid Grade Level (FKGL), Gunning Fog Index (GFI), Flesch Reading Ease Score (FRES), Simple Measure of Gobbledygook (SMOG), Automated Readability Index (ARI), Linsear Write Formula (LW), and the Coleman–Liau Index (CLI)). The rationale for using a battery of formulas rather than a single index lies in the inherent linguistic variability of AI-generated medical content. These metrics were used to determine the appropriateness of AI-generated texts for general language use and the level of cognitive comprehensibility [23,26,27].

The readability outcomes were summarized using median values along with their corresponding minimum and maximum ranges to represent the general understandability of the evaluated materials. These results were then assessed in relation to the sixth-grade reading benchmark advised by both the National Institutes of Health (NIH) and the American Medical Association (AMA). Within this framework, a FRES of 80.0 was established as the target cut-off, whereas for the remaining six readability indices, the reference level was set at a grade level of 6.0 [23,26].

### Reliability analysis methodology

The reliability of the evaluated information sources was assessed using the modified version of the DISCERN instrument [27]. This methodological tool evaluates sources using a score ranging from 0 to 5 based on five separate criteria, with higher scores indicating a higher level of reliability. The scale’s questioning items focus on five fundamental dimensions of source content: whether additional references are cited, timeliness, clarity of language, approach to controversial issues, and objectiveness of content [28]. Furthermore, the validity and reliability of assessment scales such as DISCERN and JAMA have been confirmed by previous studies in the literature [29,30].

The second criterion used to determine reliability was based on The Journal of the American Medical Association (JAMA) Criteria Set. In the analysis conducted using this set, the academic quality of the scientific studies examined was filtered according to four critical publication ethics principles: currency, attribution, authorship and disclosure [31].

The evaluation process, conducted in accordance with the JAMA criteria, relied on a binary rating method (0 or 1) to confirm the study’s eligibility for each criterion. The resulting total score, with a maximum score of 4, demonstrates the overall academic credibility of the research. A high score confirms that the study demonstrates superior adherence to these ethical standards and is therefore more credible [32].

### Content quality analysis

Two primary assessment tools are widely used in the literature to assess the quality of online health content. The Global Quality Score (GQS) was employed as the initial evaluation metric. This five-pt ordinal scale measures the overall quality of digital health content, where a rating of 1 denotes poor-quality material offering minimal patient value, and a rating of 5 signifies information that is comprehensive, trustworthy, and clinically informative. Intermediate scores are categorized as follows: 2 represents low quality and limited potential for use, 3 represents moderate quality and limited benefit, and 4 represents high quality and clinically useful content [33–35]. The reliability and validity of the GQS tool have been supported by previous scientific studies [36].

The second method is the EQIP (Ensuring Quality Information for Patients) tool, which systematically monitors the quality of medical texts. EQIP evaluates content using a 20-question questionnaire, and responses are coded as “yes,” “partially,” or “no” and scored as 1, 0.5, and 0, respectively. The final quality score is calculated as a percentage by dividing the total score by 20, removing “does not apply” items, and multiplying by 100 [37]. The resulting percentage scores categorize content quality into four main categories, from highest to lowest: 76–100% represents “well-written” content; 51–75% represents “good quality with minor issues”; 26–50% represents “significant problems in quality”; and 0–25% represents “severe problems in quality” [38].

### Statistical assessment

Data processing for the entire study was executed using IBM SPSS Statistics software, version 24.0 (IBM Corp., USA). Metrics defined as categorical variables were reported using counts and corresponding proportions, while continuously measured variables were presented utilizing medians alongside the extremes of their distribution (minimum–maximum). For evaluating differences across categorical data, we utilized the chi-square test and the Fisher’s exact test. Inter-group comparisons involving continuous variables were carried out via the Mann-Whitney U test. Agreement between two reviewers (E.O. and V.H.) was assessed using Cohen’s Kappa (κ) analysis. These reviewers performed their scores independently and were blinded to each other’s evaluations. Additionally, the reviewers were blinded to the identity of the AI models during the scoring process to prevent brand bias. Cohen’s Kappa (κ) analysis was used to assess inter-rater agreement across all 24 responses (N = 24). For any discrepancies in scoring, a final consensus was reached through joint re-evaluation. A p value of less than 0.05 was considered a statistically significant difference. In multigroup analyses, the Bonferroni adjustment was used. P value was determined by dividing by the number of comparisons made. A P value of less than 0.016 in a multigroup analysis of data with continuous values after the Bonferroni adjustment was considered a significant difference. The Bonferroni correction for a chi-square analysis is the number of comparisons being completed (i.e., row x columns = comparisons/tests). The initial scores obtained from the AI platforms and the raw dataset used for this study are provided as supporting information (see S1 File).

## Results

The most frequently used search terms by users seeking information about KOA on Google were determined using Google Trends. In total, 8 distinct KOA queries were submitted to each of the 3 AI systems, yielding 24 responses. Each response was individually assessed for readability, quality and reliability; medians were then calculated across 8 responses per model. This analysis revealed that the top three most frequently searched keywords were “osteoarthritis of knee,” “knee pain,” and “osteoarthritis knee pain.” Because the keyword “osteoarthritis of knee” was included, “Osteoarthritis in knee,” “osteoarthritis of the knee,” “osteoarthritis in the knee,” “ICD 10 osteoarthritis knee,” “osteoarthritis left knee ICD 10,” “osteoarthritis right knee ICD 10,” were removed. Because the keyword “what is osteoarthritis knee,” “knee arthritis,” “what is osteoarthritis,” was removed. Because the keyword “knee osteoarthritis exercises,” “knee exercises,” was removed. Because the keyword “osteoarthritis treatment knee,” “osteoarthritis treatment,” was removed. Because the keyword “osteoarthritis knee symptoms,” “osteoarthritis symptoms,” and “knee osteoarthritis symptoms,” were removed. The keywords “osteoarthritis icd 10,” “arthritis,” “knee joint,” “osteoarthritis hip,” “icd 10 code osteoarthritis” were removed because they were not directly related to knee osteoarthritis, which is the subject of our study.

Ultimately, the research focused on eight key keywords identified; all of these terms are detailed in Table 2.

**Table 2:** Most Frequently Searched Knee Osteoarthritis Keywords Worldwide (2004–2025, Google Trends Analysis).

Order	Keyword	Classification of Subjects According to EQIP Standards
**1**	**osteoarthritis of knee**	C&I
**2**	**knee pain**	C&I
**3**	**osteoarthritis knee pain**	C&I
**4**	**osteoarthritis treatment knee**	D&A
**5**	**what is osteoarthritis knee**	C&I
**6**	**knee osteoarthritis exercises**	D&A
**7**	**osteoarthritis knee symptoms**	C&I
**8**	**knee replacement**	D&A

EQIP: ensuring quality information for patients., C&I: Condition or illness, D&A: Discharge or aftercare.

**Fig 1:**
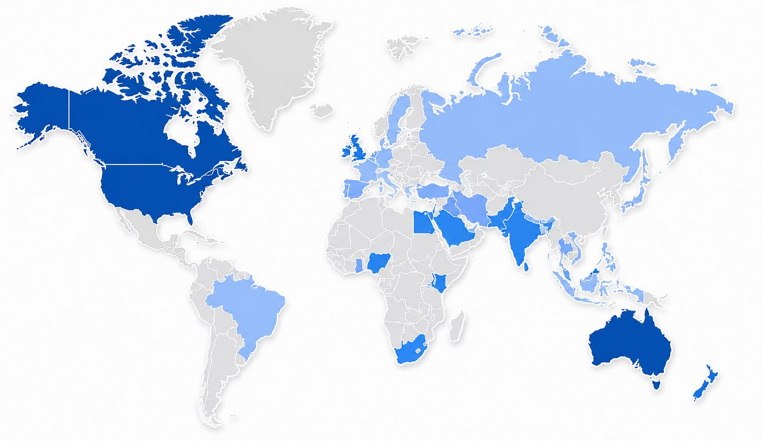
Global Patterns of Online Searches for Knee Osteoarthritis by Country (2004–2025) (Data source: Google Trends, https://www.google.com/trends).

Analysis revealed that the highest search volume for KOA originated from Puerto Rico, Malaysia, and Australia, respectively. The global geographic interest in the keyword KOA is visually presented in Fig 1.

The keywords associated with this identified KOA were submitted as query input to the designated AI chatbots (Perplexity, ChatGPT-5, and Google Gemini). The texts generated by the AI systems were analyzed to determine their readability. Each response was assessed against the 6th-grade reading level guideline established by the American Medical Association Foundation and the American Medical Association, serving as the benchmark for evaluation (Table 3).

**Table 3:** Analysis of the readability assessment of AI chatbots responses to eight frequently asked KOA inquiries, with statistical measures of text complexity relative to a 6th-grade benchmark [median (minimum–maximum)] derived from average readability scores.

CALCULATOR Statistics	ChatGPT	Gemini	Perplexity	p”	Comparison of ChatGPT versus Gemini (p)†	Comparison of ChatGPT versus Perplexity (p)†	Comparison of Gemini versus Perplexity (p)†
**FRES**	45 (41-79)	53.5 (40-69)	24.5 (7-51)	**0.001**	0.074	**0.009**	**0.002**
**GFOG**	11.9 (7.6-12.6)	10.65 (8.1 −12.5)	15 (10.40-17.10)	0.018	0.189	0.059	**0.010**
**FKGL**	9.24 (4.17-10.08)	10.23 (6.91-11.99)	13.48 (9.94-15.37)	**0.002**	0.294	**0.001**	0.014
**CLI**	14.03 (8.79-15.1)	11.45 (8.65-14.93)	17.83 (12.82 −21.27)	**0.001**	0.046	**0.012**	**0.002**
**SMOG**	8.37 (5.95-9.19)	9.74 (7.15-11.02)	11.5 (8.54-13.05)	**0.006**	0.248	**0.002**	0.031
**ARI**	11.37 (6.7-12.3)	11.84 (7.24-11.82)	15.21 (11.27-17.71)	0.018	0.563	**0.009**	0.031
**LW**	7.2 (5.99-10.44)	12.8 (8.54-15.89)	13.98 (11.16-19.81)	**< 0.001**	**0.002**	**0.001**	0.189

**Abbreviations used in this table: Artificial Intelligence (AI),** Automated Readability Index (ARI), Simple Measure of Gobbledygook (SMOG), Gunning Fog (GFOG), Flesch Reading Ease (FRES), Linsear Write (LW), Flesch–Kincaid Grade Level (FKGL), Coleman–Liau Index (CLI).

“: Krusker Wallis, “:p < 0.05 statistically significant.

†: Mann-Whitney U, †:*a p value of less than 0.016 in a multigroup analysis of data with continuous values after the Bonferroni adjustment was considered a significant difference,.*Bolded p-values indicate statistical significant.

The responses provided by AI chatbots were categorized into 3 separate functional categories (Disease Description, Symptomatology, Treatment/Management). Analyses revealed no statistically significant differences in readability, quality, and reliability between these categories (p > 0.05).

### Comparative analysis of readability levels of different artificial ıntelligence models

When the readability scores of the response texts obtained from three different AI systems were evaluated, significant differences were identified in the pairwise comparisons between these groups. These findings indicate that the understandability profiles of the information produced by different AI platforms are not homogeneous. Statistically significant differences were observed among the LLMs regarding readability and quality indices (p < 0.05). The calculated effect sizes (ε^2^) indicated a remarkably high magnitude of difference, particularly for Linsear Write (ε^2^–2.190), Flesch Reading Ease (ε^2^–1.896), and EQIP (ε^2^–2.412). The effect sizes for other parameters were as follows: ARI (ε^2^–1.153), Gunning Fog (ε^2^–1.146), FKGL (ε^2^–1.727), Coleman-Liau (ε^2^–1.872), and SMOG (ε^2^–1.479).

A pairwise analysis between ChatGPT-5 and Gemini revealed statistically significant differences in the LW (p = 0.002) readability formula, while no significant differences were found for the other metrics. The comparison between Perplexity and Gemini revealed significant differences in FRES (p = 0.002), GFOG (p = 0.010) and CLI(p = 0.002) readability formulas. Similarly, the analysis between Perplexity and ChatGPT-5 revealed significant differences (ARI p = 0.009, FRES p < 0.009, FKGL = 0.001, CLI p = 0.012, SMOG p = 0.002, LW p = 0.001) in all remaining readability formulas except GFOG (p = 0.059).

As a result of the interpretation of the obtained results, these findings suggest that ChatGPT-5 provided the most easily readable responses; this model was followed by Gemini and Perplexity, respectively (Table 3).

### Comparing AI responses to the recommended sixth grade reading standard

It was determined that there was a statistically significant deviation between the median readability values of all AI responses and the recommended sixth-grade reading level (p < 0.05), (ARI p < 0.001, FRES p < 0.001, GFOG p < 0.001, CLI p < 0.001, LW p < 0.001, FKGL p = 0.007, SMOG p = 0.007). This result demonstrates that the readability levels of the responses provided by the AI chatbots exceeded the sixth-grade readability threshold across all criteria examined (Table 3).

### Assessment results regarding content quality and source reliability

Table 4 presents a detailed overview of GQS, EQIP, DISCERN, and JAMA scores for the responses generated by all AI chatbots. A significant difference was found between the models in terms of GQS nominal classes. (*x*^*2*^ (4, *N* = 24) = 24.205, p < .001). The effect size of this difference is quite strong. (Cramer’s V = .710). The significant difference found between the models in terms of JAMA scores has a strong effect size (*x*^*2*^ (4, *N* = 24) = 18.429, p = .001) (Cramer’s V = .620). A statistically significant difference was observed between the reliability levels (mDISCERN) of the models (*x*^*2*^ (6, *N* = 24) = 34.167, p < .001). The calculated effect size was one of the highest across all metrics. (Cramer’s V = .844). The distribution of content quality according to EQIP standards shows significant differences between the models (*x*^*2*^ (2, *N* = 24) = 16.000, p < .001). The effect size of this difference is very high (Cramer’s V = .816).

**Table 4:** Ratings of AI system responses (ChatGPT-5, Gemini, Perplexity) assessed via Modified DISCERN, EQIP, GQS, and JAMA tools.

	ChatGPT-5	Perplexity	Gemini	P*
**GQS, n (%)**				**< 0.001**
**1-pt**	8(100)	0 (0)	3 (37.5)	
**2-pt**	0 (0)	0 (0)	2 (25)	
**3-pt**	0 (0)	0 (0)	2 (25)	
**4-pt**	0 (0)	5 (62.5)	1(12.5)	
**5-pt**	0 (0)	3 (37.5)	0 (0)	
**JAMA, n (%)**				**< 0.001**
**0-pt**	8(100)	0 (0)	2 (25)	
**1-pt**	0 (0)	0 (0)	4 (50)	
**2-pt**	0 (0)	6 (75)	1 (12.5)	
**3-pt**	0 (0)	2 (25)	1 (12.5)	
**m DISCERN, n (%)**				**< 0.001**
**1-pt**	7(87.5)	0 (0)	2 (25)	
**2-pt**	1(12.5)	0 (0)	3 (37.5)	
**3-pt**	0 (0)	0 (0)	3 (37.5)	
**4-pt**	0 (0)	8 (100)	0 (0)	
**EQIP, n(%)**				**< 0.001**
**Good quality with minor problems**	8(100)	0 (0)	4 (50)	
**Well written**	0 (0)	8(100	4 (50)	

**Abbreviations used in this table: pt: point, JAMA:** Journal of the American Medical Association benchmarks, EQIP: ensuring quality information for patients.

*: Chi-Square test, After Bonferroni correction; p < 0.003 for GQS, p < 0.004 for JAMA and mDISCERN, p < 0.008 for EQIP were statistically significant.

Bolded p-values indicate statistical significant.

The JAMA, DISCERN, GQS and EQIP evaluation results (median, 95% Confidence Interval (CI) (Lower limit of confidence interval- Upper limit of confidence interval)) of the answers given by ChatGPT were as follows: 1 (1–1), 1 (1–2), 1 (1–1), 60.72 (64.29–57.14). The JAMA, DISCERN, GQS and EQIP evaluation results (median, 95% Confidence Interval (CI) (Lower limit of confidence interval- Upper limit of confidence interval))of the answers given by Google Gemini were as follows; 1 (1–2), 2 (1–3), 1 (1–3), and 71.43 (77.74–59.76). The JAMA, DISCERN, GQS and EQIP evaluation results (median, 95% Confidence Interval (CI) (Lower limit of confidence interval- Upper limit of confidence interval)) of the answers given by Perplexity were as follows; 2 (2–3), 4 (4–4), 3 (3–3) and 92.86 (93.22–87.85). Statistically significant differences were detected in the JAMA DISCERN GQS and EQIP analysis in the overall analysis where all three AI chatbots were studied together.

In pairwise comparisons, Bonferroni correction was applied to chi-square tests. Perplexity scored significantly higher on the GQS (p < 0.001, Fischer’s exact test), JAMA (p < 0.001, Chi-square test), mDISCERN (p < 0.001, Chi-square test), and EQIP (p < 0.001, Fischer’s exact test) questionnaires than ChatGPT-5. Similarly, Perplexity scored significantly higher on the mDISCERN (p = 0.001, Chi-square test) questionnaire than Gemini. No statistically significant difference was found between Gemini and ChatGPT in reliability and quality surveys.

Comparisons revealed that Perplexity scored significantly higher than ChatGPT-5 in all surveys and Gemini in the Modified DISCERN assessment. Similarly, Gemini was found to score significantly higher than ChatGPT-5 in the EQIP assessment. These findings suggest that Perplexity provides the most reliable and high-quality results, however no statistical difference in reliability and quality was detected between Gemini and ChatGPT. For GQS and EQIP, the Cohen’s κ value between two reviewers were 0.742 and 0.843, respectively. For JAMA and mDISCERN scales, the Cohen’s κ values between two reviewers were 0.759 and 0.815, respectively.

### Correlation analysis

Correlation analysis and impact strengths reveal strong positive correlations between the reliability and quality surveys. There are also correlation strengths that lead to statistically significant relationships between readability indices (Table 5).

**Table 5:** Assessing the Relationship and Direction Among Study Variables Using Correlation Analysis.

	ARI	FRES	GFOG	FKGL	CLI	SMOG	LW	EQIP	GQS	JAMA	mDISC
ARI	1										
FRES	**−.71**	1									
GFOG	**.75**	**−.85**	1								
FKGL	**.94**	**−.76**	**.75**	1							
CLI	**.75**	**−.97**	**.82**	**.77**	1						
SMOG	**.94**	**−.68**	**.75**	**.96**	**.70**	1					
LW	**.42**	−.29	.14	**.60**	.27	**.54**	1				
EQIP	**.52**	**−.59**	.34	**.63**	**.58**	**.52**	**.61**	1			
GQS	**.56**	**−.47**	.37	.66*	**.46**	**.60**	**.73**	.86	1		
JAMA	**.60**	**−.43**	.34	**.71**	**.42**	**.64**	**.73**	.83	**.95**	1	
mDISC	**.61**	**−.55**	**.43**	**.72**	**.53**	**.66**	**.71**	.71	.84	.94	1

Spearman’s correlation test, Bold font indicates statistically significant correlations (p < 0.05).

**Abbreviations:** Flesch Reading Ease Score (FRES), Gunning FOG (GFOG), Flesch-Kincaid Grade Level (FKGL), Simple Measure of Gobbledygook (SMOG), Coleman-Liau Index (CLI), Automated Readability Index (ARI), Linsear Write (LW), Global Quality Score (GQS), Journal of American Medical Association (JAMA), Ensuring Quality Information for Patients (EQIP), mDISC (Modified DISCERN).

## Discussion

This study determined that the responses provided by leading AI-based chat systems (such as Perplexity, Gemini, and ChatGPT) to common queries about KOA exceed the 6th-grade reading standard recommended by the US Department of Health and Human Services (HHS) and the National Institutes of Health (NIH). It has been determined that ChatGPT-5 has the best readability compared to other AI models and Perplexity has the highest quality/reliability scores according to GQS, EQIP, DISCERN, JAMA survey results. No statistically significant difference was found between Gemini and chatGPT-5 in terms of quality and reliability. A large-scale review of the KOA content generated by these AI platforms was conducted for perceived reliability on DISCERN/JAMA tools, quality based on GQS/EQIP tools and ease of understanding (readability). Our current scientific review is one of the first comprehensive analyses to examine the answers to the most frequently asked questions about KOA generated by popular LLMs, thus contributing significantly to the relevant scientific literature pool.

eHealth literacy, introduced to the literature by Norman and Skinner in 2006, is defined as individuals’ ability to seek, understand, evaluate, and use health information through digital resources to solve health problems [39]. Individuals with this set of competencies are more informed and empowered in their own health management, which is positively associated with higher-quality health behaviors and knowledge [40,41]. Conversely, low eHealth literacy is associated with negative clinical outcomes, such as increased hospital admissions, lower use of preventive services, poorer overall health, and higher healthcare costs [42]. Many adults today have low health literacy, struggling to understand texts above a 6th-grade level. Our study results show that the responses provided by all three AI systems exceed this recommended readability level.

In the current literature, the limited number of studies evaluating online information about KOA found that the readability levels of these texts were above the recommended standard and were not of sufficient quality. A study evaluating 62 Japanese KOA-related websites on Google, Yahoo, and Bing found that the websites were difficult to understand and of poor quality [43]. Another study evaluating 20 KOA-related websites on Google and Bing found a median Flesch reading ease of 53 (range = 21−74) and a Flesch-Kincaid grade of 8 (range = 5−11). The authors noted that while many websites offered accurate and clear content consistent with basic research evidence, the quality of the information was low, with significant variations in comprehensiveness, reliability, and readability [44]. AI-based chatbots are now actively involved as patient education materials, and as such, they are a focus of KOA-related research in the scientific literature, as well as in various health topics. A study examining ChatGPT-3.5’s responses to 30 questions related to KOA noted difficulty in reading and low quality. The authors emphasized that while ChatGPT provides accurate information, it may currently be perceived as a difficult tool for patients to use. They noted that technological advancements to improve readability and presentation fidelity could make it more useful [45]. A study examining the responses obtained by asking the ChatGPT-3.5 and ChatGPT-4 versions of the 23 most common questions from patients about Platelet-Rich Plasma Therapy in KOA revealed moderate quality responses in both models. However, ChatGPT-4 reported that the quality was slightly improved by adding references from studies in databases such as PubMed to the responses. However, the authors noted that responses consistently and significantly exceeded the recommended 6th-grade reading level for PEMs and emphasized that this complexity could limit the understandability and accessibility of information for the general public, potentially limiting the effectiveness of such tools in patient education [46].

While popular LLMs technology has received considerable criticism, there are also studies in the literature demonstrating the high quality and readability of AI-generated responses. A study in which 30 of the most frequently asked questions by patients regarding “tibial osteotomy surgery for the treatment of KOA” were asked using both ChatGPT-4 and the fine-tuned ChatGPT-4, which produces more specialized, consistent, and targeted output, yielded interesting results. The authors emphasized that the fine-tuned model significantly outperformed native ChatGPT-4 in terms of response quality and readability, and that fine-tuning in future ChatGPT models could further enhance the provision of reliable and personalized information to patients [47]. Another study conducted in China found that personalized self-management guidance prepared by ChatGPT-4 for KOA patients was more efficient, accurate, personalized, comprehensive, and secure than clinician-generated guidance. The authors finally emphasized that Chat-GPT may need readability improvements to maximize patient understanding [48].

Similar to our study, significant results have been obtained in studies comparing multiple AI chatbots in the literature. In a study evaluating outcomes related to palliative care, all results were found to have readability values higher than the 6th-grade average, in line with our readability scores. Readability levels, from easy to difficult, were determined for Bard®, Copilot®, Perplexity®, ChatGPT®, and Gemini®. The same study found that mDISCERN and JAMA scores were highest for Perplexity®, while Gemini® responses had the highest GQS scores [26]. In a study on cardiopulmonary resuscitation, readability levels were determined as Bard, Perplexity, Gemini, and ChatGPT-3.5, from easy to difficult, and it was emphasized that the high number of sources found in the results of Perplexity resulted in high JAMA and DISCERN results [25]. In summary, these findings suggest that ChatGPT has gained a great reputation by becoming a very effective tool for users and authors by processing natural languages; Gemini maintains its popularity by providing the most up-to-date information that improves user search results; and Perplexity AI has gained a great reputation by providing accurate, precise, and relevant information by aggregating or summarizing sources from various sources such as websites or journals [49].

The finding that all ChatGPT-5 responses received a JAMA score of 0 suggests a notable trend in how free-tier LLMs provide information. This outcome is potentially a consequence of the model’s default configuration, which tends to provide direct answers without explicit citations or authorship attribution in standard interactions. Consequently, the observation that Perplexity yielded higher reliability scores should perhaps be interpreted as a reflection of its citation-by-design architecture. As a search-augmented engine, Perplexity is inherently structured to integrate web citations, which naturally aligns more closely with the requirements of scales like JAMA and mDISCERN. This distinction is critical; it suggests that while certain models appear more ‘reliable’ on paper, these scores may potentially reflect the model’s operational framework rather than the absolute accuracy or clinical superiority of the content itself.

Consistent with the literature, our findings underscore that while AI models provide immediate health information, they frequently fail to meet recommended readability and reliability standards for public health compliance. The inherent lack of algorithmic transparency and citation consistency across most platforms necessitates caution when using these tools for clinical decision-making. Ultimately, AI-generated content should be viewed as a supplementary resource rather than a substitute for professional medical consultation. To ensure patient safety, future AI developments must align more closely with health literacy guidelines and prioritize evidence-based accountability.

As a positive development, designed to evaluate the reliability and clinical utility of LLMs developed by OpenAI in the healthcare domain, the HealthBench benchmark offers a significant advantage by providing a multidimensional assessment that moves beyond simple factual recall. HealthBench is an opensource benchmark developed in partnership with 262 physicians who collectively have practiced in 60 countries and comprising 5,000 realistic health conversations, which is designed to address existing gaps. It uniquely measures five critical dimensions (Factual Accuracy, Reasoning Ability, Medical Consistency, Safety, and Trustworthiness)by prioritizing scenarios that reflect real-world clinical utility and using expert-curated data. However, a limitation of HealthBench is the inherent need for continuous updating to keep pace with the rapidly evolving fields of both medicine and AI model architecture, thus ensuring the test remains valid and free from data contamination [50,51].

The accessibility and quality of health information are not only concerns in English-language content but also present significant challenges in other linguistic contexts. For instance, a study evaluating Turkish internet-based patient education materials for ‘Low Back Pain’ demonstrated that such resources often exceed the average reading level of the general population, mirroring the readability barriers found in our current analysis [52]. This suggests that regardless of the language or platform used, there is a consistent global gap between the technical complexity of digital health information and the actual health literacy levels of patients.

### The role of AI in chronic disease management

AI chatbots are increasingly serving as digital health intermediaries that influence how the public accesses information regarding degenerative conditions like KOA. Currently functioning as a ‘first-opinion’ interface, these models provide 24/7 accessibility to management and treatment options. Furthermore, it is essential to discuss the multifaceted effects of the knowledge gained by patients through the internet and AI regarding the causes, pathophysiology, and treatment of their illness on treatment and decision-making outcomes. These AI systems are powered by complex mathematical models and datasets. These algorithms encompass many areas such as natural language understanding, image recognition, decision-making, problem-solving, and learning from experience [53]. However, our findings highlight a significant readability gap that limits their utility. For AI to safely assist in public health management, future developments must bridge the disconnect between technical accuracy and the practical health literacy needs of the average patient to ensure that AI-driven advice is both accessible and actionable

### Clinical ımplications of readability barriers

The failure of all evaluated AI models to meet the recommended 6th-grade readability threshold has direct clinical implications for patient safety and treatment adherence. When patients with KOA encounter complex, high-level medical terminology, they may misinterpret critical advice regarding exercise, weight management, or surgical indications. This lack of comprehension can lead to increased patient anxiety, ‘information overload,’ and the potential for misinformed self-management decisions. From a clinical perspective, if AI generated content remains too complex for the average adult, it may inadvertently increase the burden on healthcare providers, who must spend additional consultation time correcting misconceptions or simplifying AI derived jargon to ensure patient understanding.

Beyond AI models, platforms like YouTube and various online web channels remain primary health information sources, remain primary health information sources, with 80% of US users seeking medical guidance online during the pandemic [34,54,55]. While these innovative algorithms offer real-time accessibility, they often present technical jargon that exceeds the recommended readability levels for the general public. Consequently, AI-driven knowledge should be viewed as a supplementary tool that requires professional oversight to ensure patient safety and comprehension.

### Limitations

Several methodological limitations should be considered when interpreting the results of this study. First, the analysis was limited to only the 25 most frequently searched keywords related to KOA obtained from Google Trends; therefore, the inclusion of broader search terms (e.g., degenerative joint diseases or gonarthrosis) could have increased the scope and generalizability of the findings. The utilization of Google Trends for keyword selection was intended to mirror actual online health-seeking patterns rather than professional medical terminology. While prioritizing high-frequency terms related to KOA bolsters ecological validity, it is acknowledged that selecting alternative keywords, such as more technical or symptom-focused descriptors, could have shifted the content quality and readability results. Although the eight chosen keywords are widely searched across various regions, they may not fully represent the specialized informational requirements of certain patient subgroups. Employing diverse readability indices provides a robust methodological framework for evaluating linguistic difficulty; however, their primary clinical significance is their capacity to estimate general comprehension. Another limitation involves the potential for geographic variability in AI outputs. Although the queries were performed in English to capture a global perspective, the lack of VPN-based testing across different international servers means that regional localization might have influenced the specific content or readability of the responses.

While objective readability indices are valuable, they do not fully capture the subjective comprehension of diverse patient populations. A primary limitation of this study is the lack of a ‘live’ patient cohort for validation; however, since the study was conducted in a non-English speaking region (Turkey), testing local participants on English-language AI outputs would have introduced significant linguistic bias. Furthermore, translating these outputs would have invalidated the English-specific indices used for evaluation. Future research in native English-speaking populations is essential to bridge the gap between automated metrics and real-world patient understanding, ensuring that AI-driven advice is clinically safe and accessible.

Potential confounding factors such as response length, formatting, and citation structure differed across AI platforms and were not controlled, as these features are inherent to each model and reflect real-world user experience. Variations in model architecture and training updates may therefore have influenced readability, quality, and reliability outcomes.

Our findings are limited to English-language outputs generated by free public versions; they cannot be generalized to other languages or paid/professional versions of these models. Another limitation is that these AI models are constantly updated and different results may emerge in different versions, and even the same AI model may produce different answers to the same question at different times. Furthermore, the number of AI platforms included in the comparison was limited; studies examining a broader range of models could further explore the strengths and weaknesses of these technologies. The absence of hyperparameters such as temperature, which affects the randomness of the text in artificial intelligence models, and the maximum token, which affects the word/word fragment length in the answer, or the absence of a control group in the study can be considered as a limitation^48^. Another potential limitation of this study is the relatively small number of keywords (n = 8) analyzed per chatbot. While this sample size was determined through a rigorous filtration of the top 25 most frequent search terms to avoid synonyms and irrelevant queries, it may limit the statistical power of certain assessments, such as the correlation matrix. However, this focused approach ensures that the analysis remains grounded in the most clinically relevant and high-traffic inquiries used by patients in real-world scenarios. Future studies incorporating a broader spectrum of long-tail keywords or multi-language queries could further enhance the generalizability of these findings. A significant methodological limitation is the inherent stochastic nature of AI-based chatbots, which can produce varying responses to the identical query across different sessions. The results presented in this study reflect a single-point-of-contact analysis conducted on September 1, 2025. No stability spot-checks or multiple-iteration tests were performed for each keyword. While this approach effectively captures a ‘snapshot’ of the standard user experience, the lack of iterative testing means that the degree of output variability was not quantified. This snapshot nature is further compounded by the continuous updates to model architectures and training data, which may lead to different results in future evaluations. Therefore, the findings should be interpreted as a temporal assessment of the free public versions of these models rather than a definitive statement on their permanent performance. Another limitation of this study is the reliance on a single online readability calculator, which may introduce minor variations in scoring; however, this approach was maintained for internal consistency across all models, and future studies could benefit from averaging results across multiple platforms to further validate these linguistic metrics [56]. Finally, the limited data collection period to a single time period in September 2025 prevents the ability to fully track content changes and current trends over time. Designing future studies to include multiple language options and longer datasets would enhance the reliability and validity of the findings.

### Study strengths

The most significant contribution of this research is that it is a pioneering study that examines the information provided by AI-based chatbots regarding KOA not only in terms of readability, but also across multiple criteria such as accuracy, consistency, source citation, and content quality. Unlike previous literature, the evaluation of multiple common AI platforms using the same methodology has more clearly demonstrated the current capabilities and shortcomings of these technologies in health communication. This multidimensional and comparative approach provides valuable data that can guide the development of AI-enabled health information systems in both clinical practice and future academic studies.

## Conclusion

AI-based chat platforms (Perplexity, ChatGPT, Gemini) are rapidly improving their capacity to generate information on medical topics such as KOA. Our study findings show that the texts presented by the models examined in KOA are above the sixth-grade reading level, which is considered easily understandable by the general public; ChatGPT-5 offers easy-to-read answers among other models; and Perplexity offers reliable and high-quality results because it serves its answers with sources. But as a result, AI should not be the sole determinant in medical decision-making processes; rather, it should be positioned as a complementary tool that supports expert opinion but does not replace human oversight.

## Supporting information

S1 File The complete dataset (raw data) has been uploaded directly to the journal system as a Supporting Information file. (SAV)
