# Supplementary material for: A comparative analysis of readability, quality, and reliability in large language model outputs pertaining to knee osteoarthritis queries
Source: PLoS One. 2026 Jul 29;21(7):e0353355. doi: 10.1371/journal.pone.0353355 (PMC13419221; doi:10.1371/journal.pone.0353355)
Supplement: Attachment — Submitted filename: Response to Editorial Queries.docx [file pone.0353355.s003.docx]

Dear Editorial Office,

Thank you for your email and for checking our submission.

We have now updated the Data Availability Statement by adding the repository name for the uploaded SPSS/raw data files. In addition, the web archive link containing all keywords and AI chatbot-generated responses has been included in the revised manuscript.

Figure 1 is our own original visualization created using data exported from Google Trends. It is not a screenshot taken directly from the Google Trends interface. Therefore, Google Trends has been cited only as the data source.

Thank you for your consideration.

Kind regards,

Dr. Ozduran
