# Supplementary material for: A comparative analysis of readability, quality, and reliability in large language model outputs pertaining to knee osteoarthritis queries
Source: PLoS One. 2026 Jul 29;21(7):e0353355. doi: 10.1371/journal.pone.0353355 (PMC13419221; doi:10.1371/journal.pone.0353355)
Supplement: Peer review history

# Peer review: Decision Letter 0

**DOI:** 10.1371/journal.pone.0353355.r001

**Authors:** Hamza Küçük

7 May 2026

PONE-D-26-07123

A Comparative Analysis of Readability, Quality, and Reliability in Large Language Model Outputs Pertaining to Knee Osteoarthritis Queries

PLOS One

Dear Dr. Ozduran,

Thank you for submitting your manuscript to PLOS ONE. After careful consideration, we feel that it has merit but does not fully meet PLOS ONE’s publication criteria as it currently stands. Therefore, we invite you to submit a revised version of the manuscript that addresses the points raised during the review process.

Please submit your revised manuscript by Jun 21 2026 11:59PM. If you will need more time than this to complete your revisions, please reply to this message or contact the journal office at plosone@plos.org. When you're ready to submit your revision, log on to https://www.editorialmanager.com/pone/ and select the 'Submissions Needing Revision' folder to locate your manuscript file.

Please include the following items when submitting your revised manuscript:

- A letter that responds to each point raised by the academic editor and reviewer(s). You should upload this letter as a separate file labeled 'Response to Reviewers'.
- A marked-up copy of your manuscript that highlights changes made to the original version. You should upload this as a separate file labeled 'Revised Manuscript with Track Changes'.
- An unmarked version of your revised paper without tracked changes. You should upload this as a separate file labeled 'Manuscript'.

If you would like to make changes to your financial disclosure, please include your updated statement in your cover letter. Guidelines for resubmitting your figure files are available below the reviewer comments at the end of this letter.

If applicable, we recommend that you deposit your laboratory protocols in protocols.io to enhance the reproducibility of your results. Protocols.io assigns your protocol its own identifier (DOI) so that it can be cited independently in the future. For instructions see: https://journals.plos.org/plosone/s/submission-guidelines#loc-laboratory-protocols. Additionally, PLOS ONE offers an option for publishing peer-reviewed Lab Protocol articles, which describe protocols hosted on protocols.io. Read more information on sharing protocols at https://plos.org/protocols?utm_medium=editorial-email&utm_source=authorletters&utm_campaign=protocols.

As the corresponding author, your ORCID iD is verified in the submission system and will appear in the published article. PLOS supports the use of ORCID, and we encourage all coauthors to register for an ORCID iD and use it as well. Please encourage your coauthors to verify their ORCID iD within the submission system before final acceptance, as unverified ORCID iDs will not appear in the published article. *Only*  the individual author can complete the verification step; PLOS staff *cannot*  verify ORCID iDs on behalf of authors.

We look forward to receiving your revised manuscript.

Kind regards,

Hamza Küçük, PhD.

Academic Editor

PLOS One

Journal Requirements:

When submitting your revision, we need you to address these additional requirements.

1. Please ensure that your manuscript meets PLOS ONE's style requirements, including those for file naming. The PLOS ONE style templates can be found at

https://journals.plos.org/plosone/s/file?id=wjVg/PLOSOne_formatting_sample_main_body.pdf and

https://journals.plos.org/plosone/s/file?id=ba62/PLOSOne_formatting_sample_title_authors_affiliations.pdf

2. In your Methods section, please include additional information about your dataset and ensure that you have included a statement specifying whether the collection and analysis method complied with the terms and conditions for the source of the data.

3. Thank you for stating the following in your Competing Interests section: [None].

Please complete your Competing Interests on the online submission form to state any Competing Interests. If you have no competing interests, please state ""The authors have declared that no competing interests exist."", as detailed online in our guide for authors at http://journals.plos.org/plosone/s/submit-now

This information should be included in your cover letter; we will change the online submission form on your behalf.

4. We note that Figure 1 in your submission contain [map/satellite] images which may be copyrighted. All PLOS content is published under the Creative Commons Attribution License (CC BY 4.0), which means that the manuscript, images, and Supporting Information files will be freely available online, and any third party is permitted to access, download, copy, distribute, and use these materials in any way, even commercially, with proper attribution. For these reasons, we cannot publish previously copyrighted maps or satellite images created using proprietary data, such as Google software (Google Maps, Street View, and Earth). For more information, see our copyright guidelines: http://journals.plos.org/plosone/s/licenses-and-copyright.

We require you to either (1) present written permission from the copyright holder to publish these figures specifically under the CC BY 4.0 license, or (2) remove the figures from your submission:

1. You may seek permission from the original copyright holder of Figure(s) [#] to publish the content specifically under the CC BY 4.0 license.

We recommend that you contact the original copyright holder with the Content Permission Form (http://journals.plos.org/plosone/s/file?id=7c09/content-permission-form.pdf) and the following text:

“I request permission for the open-access journal PLOS ONE to publish XXX under the Creative Commons Attribution License (CCAL) CC BY 4.0 (http://creativecommons.org/licenses/by/4.0/). Please be aware that this license allows unrestricted use and distribution, even commercially, by third parties. Please reply and provide explicit written permission to publish XXX under a CC BY license and complete the attached form.”

Please upload the completed Content Permission Form or other proof of granted permissions as an ""Other"" file with your submission.

In the figure caption of the copyrighted figure, please include the following text: “Reprinted from [ref] under a CC BY license, with permission from [name of publisher], original copyright [original copyright year].”

2. If you are unable to obtain permission from the original copyright holder to publish these figures under the CC BY 4.0 license or if the copyright holder’s requirements are incompatible with the CC BY 4.0 license, please either i) remove the figure or ii) supply a replacement figure that complies with the CC BY 4.0 license. Please check copyright information on all replacement figures and update the figure caption with source information. If applicable, please specify in the figure caption text when a figure is similar but not identical to the original image and is therefore for illustrative purposes only.

The following resources for replacing copyrighted map figures may be helpful:

USGS National Map Viewer (public domain): http://viewer.nationalmap.gov/viewer/

The Gateway to Astronaut Photography of Earth (public domain): http://eol.jsc.nasa.gov/sseop/clickmap/

Maps at the CIA (public domain): https://www.cia.gov/library/publications/the-world-factbook/index.html and https://www.cia.gov/library/publications/cia-maps-publications/index.html

NASA Earth Observatory (public domain): http://earthobservatory.nasa.gov/

Landsat: http://landsat.visibleearth.nasa.gov/

USGS EROS (Earth Resources Observatory and Science (EROS) Center) (public domain): http://eros.usgs.gov/#

Natural Earth (public domain): http://www.naturalearthdata.com/

5. Please include captions for your Supporting Information files at the end of your manuscript, and update any in-text citations to match accordingly. Please see our Supporting Information guidelines for more information: http://journals.plos.org/plosone/s/supporting-information.

6. If the reviewer comments include a recommendation to cite specific previously published works, please review and evaluate these publications to determine whether they are relevant and should be cited. There is no requirement to cite these works unless the editor has indicated otherwise.

Additional Editor Comments:

Dear authors,

The peer review reports for your manuscript have been received. You may now revise your manuscript in accordance with the reviewers' comments.

Best regards.

[Note: HTML markup is below. Please do not edit.]

Reviewers' comments:

Reviewer's Responses to Questions

**Comments to the Author**

1. Is the manuscript technically sound, and do the data support the conclusions?

The manuscript must describe a technically sound piece of scientific research with data that supports the conclusions. Experiments must have been conducted rigorously, with appropriate controls, replication, and sample sizes. The conclusions must be drawn appropriately based on the data presented.

Reviewer #1: Yes

Reviewer #2: Yes

Reviewer #3: Yes

**********

2. Has the statistical analysis been performed appropriately and rigorously?

Reviewer #1: Yes

Reviewer #2: Yes

Reviewer #3: Yes

**********

3. Have the authors made all data underlying the findings in their manuscript fully available?

The PLOS Data policy requires authors to make all data underlying the findings described in their manuscript fully available without restriction, with rare exception (please refer to the Data Availability Statement in the manuscript PDF file). The data should be provided as part of the manuscript or its supporting information, or deposited to a public repository. For example, in addition to summary statistics, the data points behind means, medians and variance measures should be available. If there are restrictions on publicly sharing data—e.g. participant privacy or use of data from a third party—those must be specified.

Reviewer #1: Yes

Reviewer #2: Yes

Reviewer #3: Yes

**********

4. Is the manuscript presented in an intelligible fashion and written in standard English?

PLOS ONE does not copyedit accepted manuscripts, so the language in submitted articles must be clear, correct, and unambiguous. Any typographical or grammatical errors should be corrected at revision, so please note any specific errors here.

Reviewer #1: Yes

Reviewer #2: Yes

Reviewer #3: Yes

**********

5. Review Comments to the Author

Please use the space provided to explain your answers to the questions above. You may also include additional comments for the author, including concerns about dual publication, research ethics, or publication ethics. (Please upload your review as an attachment if it exceeds 20,000 characters)

Reviewer #1: Thank you for the opportunity to review this manuscript. The authors compare how three widely used public LLM chatbots (ChatGPT-5, Gemini 2.5 Flash, Perplexity Sonar) respond to common patient queries about knee osteoarthritis. The question is timely, the methodology follows established precedent, the dataset is openly deposited on figshare, and the conclusions are appropriately cautious. The work is publishable with modest revisions.

Major Comments

Ethics statement. The waiver justification ("as in previous studies") is too brief. Please confirm explicitly that no human participants or identifiable data were involved, and cite the institutional framework supporting the waiver.

Sample size. With n = 8 queries per chatbot (24 responses total), the Spearman correlation matrix in Table 5 is underpowered. Please state sample sizes for each test in the Methods, flag the correlations as exploratory, and consider Fisher's exact test throughout Table 4 given the zero cells.

Reproducibility. Each score reflects a single stochastic AI output. Please state whether any stability spot-check was performed; if not, sharpen this as a limitation.

Inter-rater reliability. Cohen's κ values are reported, but please clarify whether reviewers scored independently and blinded, how disagreements were resolved, and on how many responses κ was calculated.

JAMA = 0 for all ChatGPT-5 responses (Table 4) is striking and deserves explicit interpretation. Was this driven by the free-tier default suppressing citations? The "Perplexity is more reliable" headline is partly an artifact of its citation-by-design architecture rather than information quality per se — please state this directly.

Minor Comments

Abstract: Awkward phrasing on line 54; consider adding median scores for headline metrics.

Typos: "literatüre" (lines 96, 230); "COA" should be "KOA" (line 549); "scores24" merged with reference (line 462); "PEMs" used before definition (line 130).

Methods: Specify which Perplexity Sonar variant was used (Sonar, Sonar Pro, or Sonar Reasoning). Cite the "previous studies" referenced on line 151. Clarify whether the three functional categories (line 181) were pre-specified.

Table 1: Grammatical inconsistencies (e.g., "the default mode is use"). Please tidy.

Table 3: Column labelled P" is ambiguous; bold only significant cells.

Table 4: The rows "Good quality with minor problems" / "Well written" appear duplicated at top and bottom — layout error to correct.

Table 5: Cell formatting has broken in the PDF; please ensure legibility in the typeset version.

Discussion: The "Paradigm Shift" subsection (lines 492–501) is speculative and not supported by the study data; consider tightening. Add one paragraph of practical clinical implications of the sub-6th-grade readability failure.

References: Verify Ref 1 year (2013 vs 2023 based on DOI); Ref 48 formatting is broken; check PLOS ONE style compliance throughout.

Language: A light copy-edit by a native English speaker would polish the text.

Summary

A competent, useful contribution to the growing literature on AI chatbot performance in patient-facing health information. Core findings are credible. Concerns are confined to sample-size transparency, interpretation of the JAMA-zero result, and tidying of tables and language. None are blocking.

I recommend Minor Revision.

Reviewer #2: The manuscript presents an interesting and timely evaluation of readability, quality, and reliability of information generated by large language models for knee osteoarthritis–related queries. The topic is relevant given the increasing use of AI systems by patients seeking medical information. The manuscript is generally well organized and technically sound; however, several points could be improved to strengthen the study.

1. Sample Size and Prompt Diversity

The study evaluates responses generated from only eight search queries, resulting in 24 outputs across three AI systems. While this approach provides preliminary insights, the limited number of prompts may restrict the generalizability of the findings. The authors should acknowledge this limitation more explicitly and discuss how including a larger set of queries or repeated prompt sampling could enhance future research.

2. Prompt Standardization

The methodology would benefit from a clearer description of how prompts were structured and submitted to each AI system. It would be helpful to clarify whether the prompts were standardized across models and whether repeated runs were performed to account for variability in AI-generated responses. Providing the exact prompt template (possibly as supplementary material) would improve methodological transparency.

3. Model Version and Reproducibility

Additional information regarding the specific model versions, access method (web interface or API), and the exact date of data collection should be provided. Given the rapidly evolving nature of large language models, these details are essential for reproducibility.

4. Statistical Reporting

Although appropriate statistical analyses were conducted, the results section could be strengthened by reporting effect size measures and confidence intervals where applicable. This would provide a clearer interpretation of the magnitude of differences between the evaluated systems.

5. Justification for Multiple Readability Metrics

The authors employ several readability indices (e.g., FKGL, SMOG, ARI, CLI). A brief justification for selecting multiple readability formulas and their relevance in evaluating patient-oriented health information would improve the methodological rationale.

6. Clinical Relevance

The discussion section could be expanded to better highlight the clinical implications of the findings. In particular, the authors may elaborate on how the readability and reliability of AI-generated content may influence patient education, health literacy, and patient–physician communication.

7. Language and Minor Editorial Issues

Minor grammatical inconsistencies and repetitive phrasing are present in several sections of the manuscript. A careful language revision would improve readability and overall clarity.

8. Limitations

The limitations section could be expanded to include the single time-point evaluation of AI systems, possible variability in chatbot responses, and the rapidly evolving nature of large language models.

Overall, the manuscript addresses an important and emerging topic, and the suggested revisions would further strengthen its methodological clarity and clinical relevance.

Reviewer #3: Dear Editor and Authors,

I have carefully read the authors' study evaluating the content of three different artificial

intelligence platforms related to knee osteoarthritis in terms of readability, reliability, and

quality.

With the advancement of technology, the article focuses on the originality, timeliness, and

impact on public health of the information on this subject. I can say that the authors have done

a wonderful job and that it is an enjoyable article for the journal's readers. It is recommended

that the following elements be carefully considered and appropriately revised before

publication of the article:

ABSTRACT

• The explanatory version of KOA is written, but the one for OA is not. Please write the

explanatory version of OA as well.

• Some keywords start with a capital letter, some with a lowercase letter. A standardization is

needed.

INTRODUCTION

• The introduction section is strengthened with numerical data. It can be considered a strong

article introduction.

• Please correct the error(typo) in the text ""hallucinations" in the literatüre [10]."

• “AI code of conduct developed by the US-EU Trade and Technology Council would be

beneficial to all countries, whether developing or developed [13].”

Write the full versions of the abbreviations here.

• “Similarly, findings from the U.S. Department of Education’s National Center for Education

Statistics indicate that more than half (approximately 54%) of Americans between the ages of

16 and 74 read at a level below the sixth grade.” Sometimes US, sometimes U.S., a

standardization is needed.

• Knee OA (OA) accounts for approximately four-fifths of the global OA burden, with

prevalence rising with obesity and advancing age [2].

Knee osteoarthritis (KOA) is one of the most frequently searched health topics online, yet the

accuracy, readability, and reliability of information…

Ensure standardization for abbreviations in many similar cases within the article.

• The authors' mention of the increasing role of social media platforms in promoting medical

literature is very valuable. In addition, the perspective presented in other studies in the

literature regarding the importance of "Altmetric" data, which complements the shortcomings

of traditional citations, is quite valuable; furthermore, the emphasis that the authors can make

on the historical development of the literature and the presentation of the most interactive

studies within a bibliometric framework will increase the scientific depth of the article. I

request that the authors strengthen the link between social media interaction and academic

visibility in light of this current bibliometric data, emphasizing the importance of bibliometric

analyses that reveal the development of the literature with modern data.

• Also discuss the effects of the knowledge gained by patients through the internet and AI on

the causes, pathophysiology, and treatment of their illness on treatment and decision-making

outcomes.

• It is known that artificial intelligence and social media platforms can yield very valuable

results even in pandemic or major crisis situations for humanity. Discuss this situation.

MATERIALS & METHODS

• “While the English language was used to ensure global relevance, it is noted that LLM

responses may vary slightly based on regional server nodes and localized algorithms.”

Write the full name of LLM.

• Explain why you chose these three AI models and why you didn't choose the others. • In

your research methodology, clearly state whether you've signed out of your Google or other

accounts and whether you've used Google incognito mode. Otherwise, remember that videos

in your language may be more likely to appear based on the algorithm. • Only one calculator

was used when calculating readability. Sometimes this gives incorrect values. Either use

calculators from two different websites and average the results, or add this to the limitations

by referring to the following article.

DISCUSSION

• What about online information/AI in languages other than English? For example, how do

the reliability, quality, and readability results of these findings appear in a study in a different

language? Discuss this situation.

• AI-powered innovative developments have been mentioned. YouTube, which remains a

popular application, and online web channels are among the leading sources of health-related

information for users. Discuss this situation.

**********

6. PLOS authors have the option to publish the peer review history of their article (what does this mean?). If published, this will include your full peer review and any attached files.

If you choose “no”, your identity will remain anonymous but your review may still be made public.

**Do you want your identity to be public for this peer review?**  For information about this choice, including consent withdrawal, please see our Privacy Policy.

Reviewer #1: No

Reviewer #2: No

Reviewer #3: No

**********

[NOTE: If reviewer comments were submitted as an attachment file, they will be attached to this email and accessible via the submission site. Please log into your account, locate the manuscript record, and check for the action link "View Attachments". If this link does not appear, there are no attachment files.]

To ensure your figures meet our technical requirements, please review our figure guidelines: https://journals.plos.org/plosone/s/figures

You may also use PLOS’s free figure tool, NAAS, to help you prepare publication quality figures: https://journals.plos.org/plosone/s/figures#loc-tools-for-figure-preparation.

NAAS will assess whether your figures meet our technical requirements by comparing each figure against our figure specifications.

# Author comment: Author response to Decision Letter 1

**DOI:** 10.1371/journal.pone.0353355.r002

22 May 2026

Added in the files

Attachment Submitted filename: Response to Editorial Queries.docx

# Peer review: Decision Letter 1

**DOI:** 10.1371/journal.pone.0353355.r003

**Authors:** Hamza Küçük

23 Jun 2026

A Comparative Analysis of Readability, Quality, and Reliability in Large Language Model Outputs Pertaining to Knee Osteoarthritis Queries

PONE-D-26-07123R1

Dear Dr. Ozduran,

We’re pleased to inform you that your manuscript has been judged scientifically suitable for publication and will be formally accepted for publication once it meets all outstanding technical requirements.

Within one week, you’ll receive an e-mail detailing the required amendments. When these have been addressed, you’ll receive a formal acceptance letter and your manuscript will be scheduled for publication.

An invoice will be generated when your article is formally accepted. Please note, if your institution has a publishing partnership with PLOS and your article meets the relevant criteria, all or part of your publication costs will be covered. Please make sure your user information is up-to-date by logging into Editorial Manager at Editorial Manager® and clicking the ‘Update My Information' link at the top of the page. For questions related to billing, please contact billing support.

If your institution or institutions have a press office, please notify them about your upcoming paper to help maximize its impact. If they’ll be preparing press materials, please inform our press team as soon as possible -- no later than 48 hours after receiving the formal acceptance. Your manuscript will remain under strict press embargo until 2 pm Eastern Time on the date of publication. For more information, please contact onepress@plos.org.

Kind regards,

Hamza Küçük, PhD.

Academic Editor

PLOS One

Additional Editor Comments (optional):

Dear authors, I congratulate you on your effort.

Best regards.

Reviewers' comments:

Reviewer's Responses to Questions

**Comments to the Author**

1. If the authors have adequately addressed your comments raised in a previous round of review and you feel that this manuscript is now acceptable for publication, you may indicate that here to bypass the “Comments to the Author” section, enter your conflict of interest statement in the “Confidential to Editor” section, and submit your "Accept" recommendation.

Reviewer #2: All comments have been addressed

Reviewer #3: All comments have been addressed

**********

2. Is the manuscript technically sound, and do the data support the conclusions?

The manuscript must describe a technically sound piece of scientific research with data that supports the conclusions. Experiments must have been conducted rigorously, with appropriate controls, replication, and sample sizes. The conclusions must be drawn appropriately based on the data presented.

Reviewer #2: Yes

Reviewer #3: Yes

**********

3. Has the statistical analysis been performed appropriately and rigorously?

Reviewer #2: Yes

Reviewer #3: Yes

**********

4. Have the authors made all data underlying the findings in their manuscript fully available?

The PLOS Data policy requires authors to make all data underlying the findings described in their manuscript fully available without restriction, with rare exception (please refer to the Data Availability Statement in the manuscript PDF file). The data should be provided as part of the manuscript or its supporting information, or deposited to a public repository. For example, in addition to summary statistics, the data points behind means, medians and variance measures should be available. If there are restrictions on publicly sharing data—e.g. participant privacy or use of data from a third party—those must be specified.

Reviewer #2: Yes

Reviewer #3: Yes

**********

5. Is the manuscript presented in an intelligible fashion and written in standard English?

PLOS ONE does not copyedit accepted manuscripts, so the language in submitted articles must be clear, correct, and unambiguous. Any typographical or grammatical errors should be corrected at revision, so please note any specific errors here.

Reviewer #2: Yes

Reviewer #3: Yes

**********

6. Review Comments to the Author

Please use the space provided to explain your answers to the questions above. You may also include additional comments for the author, including concerns about dual publication, research ethics, or publication ethics. (Please upload your review as an attachment if it exceeds 20,000 characters)

Reviewer #2: Önceki inceleme turunda ortaya çıkan yorumlara yanıt verdiğiniz için teşekkür ederiz. Revizyon edilen makale, metodolojik şeffaflık, sınırlamaların tartışılması ve genel açıklık açısından önemli ölçüde iyileştirilmiştir.

Yazarlar, hızlı standartlaşma, tekrarlanabilirlik ve klinik önem konusundaki temel endişeleri yeterince ele almışlardır. El yazması artık çalışma metodolojisinin daha net bir tanımını sunuyor ve bulguların güçlü ve sınırlı yanlarını daha dengeli bir şekilde tartışıyor.

Bence makale bilimsel olarak doğru, analizler uygun ve sonuçlar sunulan verilerle destekleniyor. Başka önemli yorumum yok ve el yazmasının mevcut haliyle yayımlanmaya uygun olduğuna inanıyorum.

Reviewer #3: The authors' revised manuscript has greatly improved the overall quality of the paper. All of my previous comments have been adequately addressed, and I have no additional suggestions. I believe the manuscript is suitable for publication in its current form.

**********

7. PLOS authors have the option to publish the peer review history of their article (what does this mean?). If published, this will include your full peer review and any attached files.

If you choose “no”, your identity will remain anonymous but your review may still be made public.

**Do you want your identity to be public for this peer review?**  For information about this choice, including consent withdrawal, please see our Privacy Policy.

Reviewer #2: No

Reviewer #3: No

**********

# Editor report: Acceptance letter

**DOI:** 10.1371/journal.pone.0353355.r004

**Authors:** Hamza Küçük

PONE-D-26-07123R1

PLOS One

Dear Dr. Ozduran,

I'm pleased to inform you that your manuscript has been deemed suitable for publication in PLOS One. Congratulations! Your manuscript is now being handed over to our production team.

At this stage, our production department will prepare your paper for publication. This includes ensuring the following:

* All references, tables, and figures are properly cited

* All relevant supporting information is included in the manuscript submission,

* There are no issues that prevent the paper from being properly typeset

You will receive further instructions from the production team, including instructions on how to review your proof when it is ready. Please keep in mind that we are working through a large volume of accepted articles, so please give us a few days to review your paper and let you know the next and final steps.

Lastly, if your institution or institutions have a press office, please let them know about your upcoming paper now to help maximize its impact. If they'll be preparing press materials, please inform our press team within the next 48 hours. Your manuscript will remain under strict press embargo until 2 pm Eastern Time on the date of publication. For more information, please contact onepress@plos.org.

You will receive an invoice from PLOS for your publication fee after your manuscript has reached the completed accept phase. If you receive an email requesting payment before acceptance or for any other service, this may be a phishing scheme. Learn how to identify phishing emails and protect your accounts at https://explore.plos.org/phishing.

If we can help with anything else, please email us at customercare@plos.org.

Thank you for submitting your work to PLOS ONE and supporting open access.

Kind regards,

PLOS ONE Editorial Office Staff

on behalf of

Dr. Hamza Küçük

Academic Editor

PLOS One
